# Soil Microarthropods and Soil Health: Intersection of Decomposition and Pest Suppression in Agroecosystems

**DOI:** 10.3390/insects10120414

**Published:** 2019-11-20

**Authors:** Deborah A. Neher, Mary E. Barbercheck

**Affiliations:** 1 Department of Plant and Soil Science, University of Vermont, 63 Carrigan Drive, Burlington, VT 05405, USA; 2 Department of Entomology, Pennsylvania State University, 501 ASI Building, University Park, PA 16802, USA; meb34@psu.edu

**Keywords:** microarthropods, decomposition, nutrient mineralization, multi-channel feeding, microbial grazer, alternate prey, detrital shunting

## Abstract

Two desirable functions of healthy soil are nutrient cycling and pest suppression. We review relevant literature on the contributions of soil microarthropods to soil health through their intersecting roles in decomposition and nutrient cycling and direct and indirect suppression of plant pests. Microarthropods can impact soil and plant health directly by feeding on pest organisms or serving as alternate prey for larger predatory arthropods. Indirectly, microarthropods mediate the ability of crop plants to resist or tolerate insect pests and diseases by triggering induced resistance and/or contributing to optimal nutritional balance of plants. Soil fauna, including microarthropods, are key regulators of decomposition at local scales but their role at larger scales is unresolved. Future research priorities include incorporating multi-channel omnivory into food web modeling and understanding the vulnerability of soil carbon through global climate change models.

## 1. Introduction

Soil is a multicomponent, multifunctional system that affects the structure and functioning of arable ecosystems through the activities of diverse soil-dwelling organisms interacting with the abiotic environment [1]. These activities are critical to ecological processes that decompose soil organic matter (SOM) to release or immobilize nutrients, stabilize soil by forming aggregates, and promote plant growth [2,3,4,5]. Plants benefit from interactions with soil microfauna by way of protection from pathogens and pests, attraction of beneficial insects, and increased tolerance to abiotic and biotic stress [6,7].

The U.S. Department of Agriculture Natural Resources Conservation Service (USDA-NRCS) defines soil health as the “continued capacity of soil to function as a vital living ecosystem that sustains plants, animals, and humans” [8]. Soil health integrates the physical, chemical, and biological characteristics, distinguished by emphasis on biological properties such as biodiversity, food web structure and ecosystem function [9,10]. A great diversity of organisms inhabit healthy soils in managed and unmanaged ecosystems, where they support ecosystem multi-functionality, suggesting that soil biodiversity is a key factor in regulating the functioning of ecosystems [11].

Soil organisms are comprised of taxa from diverse phyla. Our focus here is on one group of fauna, soil microarthropods. Size class is a practical method to classify fauna. There are three groups defined by body width: microfauna (<0.1 mm), mesofauna (0.1–2 mm) and macrofauna (>2 mm) [7]. Among the soil fauna, microarthropods comprise a large proportion of the meso- and macrofauna and play a long-recognized role in litter transformation and nutrient cycling [12,13]. Collembola (springtails) and Acari (mites) are ubiquitous among the soil microarthropods living in the litter/humus boundary and in the mineral soil profile, occurring in virtually all terrestrial habitats throughout the world [14]. Other microarthropod taxa include the Isopoda, Myriapoda, and Insecta. Protura, Diplura, and Pauropoda are usually less abundant or infrequently detected. Even so, their activities contribute to functions of the microarthropod community with the relative contribution of biotic groups to decomposition changing with disturbance intensity and other environmental factors [15].

Two desirable functions of healthy soil are nutrient cycling and pest suppression. Here, we review relevant literature on the contributions of soil microarthropods to soil and plant health through their intersecting roles in decomposition and nutrient cycling and direct and indirect suppression of plant pests. Other reviews also provide an overview of earlier works on the roles and diversity of soil microarthropods in agricultural systems [10,11,12,16,17,18,19].

## 2. Soil Health at the Intersection of Decomposition and Pest Suppression

Historically, theories about factors that control herbivorous arthropod populations focused on above-ground interactions among herbivores, their natural enemies, and plants, e.g., Feeny [20], Root [21]. More recent attention has focused on how below-ground interactions among plants, arthropods and other biota integrate to induce systemic effects [22,23,24,25]. Interactions between soil-dwelling organisms and plants not only affect plant growth and diversity, but these interactions can cascade to impact higher above- and below-ground trophic groups, e.g., herbivores, parasitoids, hyperparasitoids, and pollinators [1,23,24,26,27]. Both direct and indirect interactions with soil organisms affect plant health and above-ground communities [1]. Microarthropods can impact soil and plant health directly by feeding on pest organisms or serving as alternate prey for larger predatory arthropods. Indirectly, microarthropods mediate the ability of crop plants to resist or tolerate insect pests and diseases by triggering induced resistance and/or optimizing the nutritional balance of plants [28]. It is likely that multiple mechanisms and types of interactions contribute to the effects of microarthropods on pest suppression and plant protection among agricultural ecosystems. The hypothesized mechanisms are not mutually exclusive and likely complement each other.

## 3. Effects on Plant Nutrition, Nutrient Balance, and Soil and Plant Health

### 3.1. Plant Nutrition

Plants fuel soil food webs through rhizodeposits, such as root exudates, and surface accumulation of dead organic matter [29]. Root exudates provide a source of carbon (C) that passes directly through rhizosphere microorganisms. These inputs can modify existing C flows within the food web, including CO_2_ efflux from the soil and litter decomposition [30]. Harvest of crops, crop phenology, and abiotic stress all affect the quality and quantity of root exudates into soil [31]. Exudates attract and support microbes that, in turn, become prey for mesofauna [32]. Grazing activity by microarthropods increases the efficiency of nutrient mineralization above that of microbes alone. The microbial loop hypothesis describes this tritrophic process as compensatory grazing because decomposition is driven by more than simply microbes [33,34,35]. Likewise, soil faunal activity modifies their environment, which affects microbial decomposition and alters pore networks and, thus, water/air dynamics [36].

Decomposition is the physical breakdown and biochemical transformation of dead organic material into simpler organic and inorganic molecules. Decomposition of organic materials is fundamental to nutrient cycling within ecosystems, influencing plant health and productivity, species composition and carbon storage [37]. Litter decomposition and soil organic matter (SOM) stabilization may affect other soil properties such as sorption, nutrient availability, pH, redox potential, and water holding capacity. These soil properties directly or indirectly support many essential ecosystem services including plant production, clean water, flood protection and climate regulation [38].

Detritivorous microarthropods fragment or comminute detritus, creating smaller particles that increase surface area for microbial colonization and increase moisture levels of the substrate, thus stimulating microorganism activity. Regulating the process of microbial decomposition in a controlled and continuous manner reduces the risk of nutrient loss from agroecosystems [10,39]. Microbial decomposition is often greater in litter that included, rather than excluded, faunal feeding and usually results in a short-term increase in microbial activity that decreases in the long term, helping to stabilize SOM [40]. In addition, microarthropods can alter the nutrient availability of soil, and plants can change their biomass allocation patterns according to the form and status of soil nutrients. Microarthropods can impact plant root-to-shoot ratio by altering soil nutrient availability [41]. Coincidently, comminution also disperses fungal spores, bacteria, and particles throughout the soil profile, mixing organic and mineral fractions [40].

Microarthropod ingestion of living plant materials, detritus, and adhering organisms converts energy into microarthropod biomass and, eventually, unassimilated material into feces [40]. The feces chemistry of microarthropods differs from the food ingested [10]. For example, feces of Collembola contained greater than 40 times more NO_3_−nitrogen than the fungi and algae that they had fed upon [42]. Chemistry of the feces of the mite, *Scheloribates moestus*, had a greater relative abundance of polysaccharides and phenols and a lower relative abundance of lignin compared to corn litter prior to assimilation [35]. These shifts in chemistry affect the quality (C:N ratios) of feces [40]. Although feces provide surface area subject to colonization by microorganisms [3], a change in carbon chemistry can affect rates of decomposition. Fauna are also involved in dung decomposition and incorporation. For example, three families of the collembolan order Arthopleona and five families and three genera of Oribatida mites were found on patches of cattle dung [43]. Although significant in some systems, the role of fecal material in decomposition has yet to be included in global carbon models.

### 3.2. Effects on Nutrient Balance

The chemical composition of soil and plants, and their interaction, affect both behavioral and developmental responses in herbivorous insects [28,44,45]. The mineral balance hypothesis [46] posits that pest problems are linked to disturbances in the nutritional balances of crop plants and destruction of soil life. Excessive soluble nitrogen (N) in soil increases cellular N, ammonia and amino acids in plants, resulting in temporary accumulation of free N, sugars, and soluble amino acids that promote the growth and multiplication of insect pests and diseases. Chaboussou [46] considered biologically healthy soil as fundamental for a balanced uptake of mineral nutrients by the plant, especially micronutrients, and that a lack of micronutrients inhibits protein synthesis and leads to a build-up of nutrients in plant tissue that promote pests and disease.

Similarly, experiments demonstrating the effects of soil fertility management on attraction of an herbivore to maize, suggest that SOM and microbial activity supports a buffering capability to soil that can help maintain nutrient balance in plants [28]. In addition, soils with high SOM content and biodiversity have improved capacity to absorb and store water and, thus, reduce water stress. Water stress increases susceptibility to pests, hypothetically by restricting protein synthesis [46] that, consequently, increases soluble N in foliage making tissues more nutritious to many pests [47]. Phelan [28] suggested that an optimal nutrient balance could be achieved by managing soil to maintain high SOM to support abundant and diverse communities of soil macro- and microbiota. Diverse communities support plant health by increasing resistance to herbivory, both by appropriate primary metabolism and production of secondary defense compounds. Phelan further hypothesized that depauperate soils lack biologically-based buffering capacity, hence, creating imbalances in the ratio of certain mineral nutrients. These imbalances result in rapid plant growth with impaired primary and/or secondary metabolisms, therefore, compromising the ability of plants to resist or tolerate insect damage. Further, inefficient biochemical pathways in such plants lead to an accumulation of simple sugars, free amino acids and peptides, providing an enriched diet for arthropod herbivores. In laboratory experiments, any deleterious effects of enriching soil on herbivorous insects were at least partially plant-mediated [48,49]. Consistent with the mineral balance hypothesis, the mineral content of plant leaves explained 40–57% of the variation in Colorado potato beetle populations observed in field plots [48].

### 3.3. Enhanced Plant Tolerance or Resistance to Pests through Induced Resistance

Negative soil-based effects on herbivore fitness support the idea of soil microbe- and microarthropod-mediated effects on plant health. These relationships have been confirmed under laboratory, greenhouse, and field conditions in the absence of natural enemies and other factors that may affect pest arthropods [50,51,52]. The production of both direct and indirect systemic plant defenses is dependent on nutrient uptake by roots [25,53,54]. Feeding activities in the detrital food web stimulate nutrient turnover, plant nutrient acquisition, and plant performance, thereby indirectly influencing above-ground herbivores and plant pathogens [55]. Soil activity-associated changes in the production and distribution of carbon compounds from photosynthetic activity, or the production and mobilization of secondary metabolites, directly or indirectly protect the plant from foliar herbivores and pathogens [56,57,58]. The term “induced resistance” is a generic term for the induced state of resistance in plants triggered by biological or chemical inducers that protect non-exposed plant parts against future attack by pathogenic microbes and herbivorous insects. Induced systemic resistance (ISR) to pathogens and pests results from direct and indirect plant defenses. Exposure of roots to herbivory or to certain microbes, both pathogenic and non–pathogenic, can induce plant development of ISR against a broad range of arthropod pests and pathogens [23,25,50,53,54,59].

Expression of induced resistance is mediated by complex signaling networks that are regulated by the plant hormones jasmonic acid (JA) and salicylic acid (SA). Induction of the JA pathway may result in the production of proteinase inhibitors, defense-related volatile compounds, secondary metabolites [60,61], active phenolics and phytoalexins [62], insect repellents [63], and natural enemy attractants [64]. Although there are exceptions, JA pathways are associated with defense against necrotrophic pathogens, symbiotic fungi, and chewing insect herbivores. JA is also the main hormone that regulates a switch from growth to defense through positive and negative crosstalk with other plant hormones [65]. The JA signaling triggers the emission of complex blends of volatile organic compounds that attracts parasitoids or other natural enemies of herbivores [66,67]. The SA pathway is associated with defense against biotrophic pathogens and phloem- and cell-content-feeding arthropods that do not cause extensive cellular damage [50,52,68]. Many of the signals that trigger plant defense responses are similar or identical in plant interactions with pathogenic and beneficial microbes [69]. Additionally, the JA and SA pathways may interact antagonistically or synergistically to fine-tune defense responses [70].

In addition to phytohormone-mediated defenses, plants possess chemical defenses comprised of metabolites that represent a barrier to herbivory. These chemical defenses can be constitutive or induced by herbivory or other stresses [60,61]. Below-ground herbivory influences the dynamics of root and rhizosphere microbial community assemblages, changing root metabolites and chemical elements. These changes can cascade to changes in microbial community diversity [71]. Soil organism-plant interactions can influence the concentration or composition of defensive plant secondary metabolites [25], resulting in local or systemic effects on herbivory and plant growth [54].

As a significant component of the below-ground ecosystem, microarthropods can influence plant defenses and plant health directly by feeding on plant roots and indirectly by feeding on specific microorganisms associated with roots [41,72,73]. Root herbivory can influence plant carbon sources and rhizosphere chemistry that, in turn, modifies microbial abundance and community physiological profiles in the rhizosphere [71]. For example, grazing on mycorrhizal fungi by collembolans can support growth of soil bacteria by lowering fungal biomass and, consequently, making more resources accessible to bacterial communities [74]. Although previously believed otherwise, collembolans can be selective feeders. For example, when offered three choices of fungi, *Folsomia fimetaria* chose *Alternaria alternata* over *Fusarium oxysporum* and generally avoided *Trichoderma viride* [75]. Oribatid mites prefer fruiting species (e.g., *Cladosporium*, *Alternaria*, *Ulocladium*) and fungal species with dark mycelia pigmented [76]. In a grassland experiment, soil microarthropods, including the predatory mite, *Hypoaspis aculeifer*, and three springtail species, *Proisotoma minuta*, *Folsomia candida*, and *Sinella curviseta*, substantially altered the composition, but not biomass, of soil microbial communities [41]. Changes in the microbial communities associated with the presence of microarthropods altered plant seedling establishment, below-ground biomass, and biomass allocation patterns. These authors suggest that these effects were due to both direct impacts on plants by herbivory and indirect impacts by feeding on and changing the composition of soil microbial communities.

### 3.4. Predation

Biological control of pests by natural enemies is a key ecosystem service in both unmanaged and managed systems that has been conservatively estimated to have a value in agricultural systems in the US of $4.5 billion annually [77,78]. Even through natural control of pest organisms is a critical and well-studied ecosystem service in agroecosystems, the extent of the contribution of soil microarthropods in regulating pest populations is poorly known. Numerous soil microarthropods function primarily as predators. For example, several mite taxa within the orders Prostigmata and Mesostigmata are voracious and agile predators with a broad feeding range on many different organisms, including collembolans, other soil mites, nematodes, leaf-miners, thrips, small flies, enchytraeids, and insect larvae and eggs [19,79,80,81]. Many mesostigatid mite (Acari: Mesostigmata) species feed on nematodes and are considered top predators in the mesofaunal food web [80]. Predaceous mites can be important in reducing the densities of eggs and larvae of the maize pest, *Diabrotica* spp. (Coleoptera: Chrysomelidae) under field conditions [82,83]. For example, *Tyrophagus putrescentiae* (Sarcoptiformes: Acaridae) consumed eggs of *D. undecimpunctata howardi* in the field, detecting the eggs from up to 8 cm away [84].

Nematodes are an important component of the diet of soil microarthropods, including mites, Collembola and insects [19,81,85,86,87]. Stable isotope analyses indicate that several putative detritivorous oribatid mites (Acari: Oribatida) are more likely to be predators or scavengers than detritivores [85,88]. A field experiment confirmed that oribatid mite species including *Liacarus subterraneus*, *Platynothrus peltifer* and *Steganacarus magnus* prey intensively on nematodes [85] and can even damage cysts of the plant-parasitic nematode, *Heterodera* sp. [89]. Collembola can also be significant predators of nematodes, demonstrated in laboratory and greenhouse experiments but also observed feeding on a targeted nematode species in the field [81,90]. Using molecular markers, Read et al. [81] detected nematode-feeding by the collembolans, *Isotoma viridis* and *Isotomurus palustris*. The collembolan, *Onychiurus armatus*, perforated the cysts of *Heterodera cruciferae* and consumed the nematodes within the cysts [89].

### 3.5. Support of Natural Enemies of Pests as Alternate Prey

In addition to acting as predators or omnivorous multi-channel feeders, microarthropods can serve as prey items for predatory epigeal and euedaphic macroarthropods. The transfer process of detrital energy and nutrients to higher trophic levels is coined “detrital infusion” or “detrital shunting” [91]. A greater concentration of organic matter in soil moderates moisture and supports detritivorous alternate prey for soil-dwelling and foliar natural enemies [92,93]. In agroecosystems, the application of organic soil fertility inputs, e.g., compost or manures, is associated positively with the abundance of predatory arthropods [94]. Feeding on microarthropods by predatory arthropods, especially generalist predators, such as carabid beetles and spiders, can support and increase populations even in absence of herbivorous prey [92,93,95]. For example, the predatory mite, *Amblyseius swirskii*, controlled western flower thrips better in the presence of alternate prey astigmatic mites, *Tyrophagus putrescentiae* and *Carpoglyphus lactis*, than in their absence [96].

Collembola, especially, are considered as important alternate prey for some generalist predators when pests are scarce [97,98]. Small spiders rely on Collembola as a key prey resource, thus providing a trophic link between detrital and grazing food webs in some cropping systems [99]. The availability of a variety of alternate prey sources may stabilize predator densities by reducing intraguild predation and by helping to sustain and retain generalist predators within crops when target pests are absent or at low density, although negative effects of alternate prey on predation of target pests has also been observed [100,101]. For example, increasing densities of isotomid and entomobryid collembolans in wheat fields resulted in increases in the aphid population due to predatory cursorial spiders and carabid and staphylinid beetles switching from feeding on aphids to feeding on decomposers [102].

### 3.6. Multi-Channel Feeding

**Figure 1:**
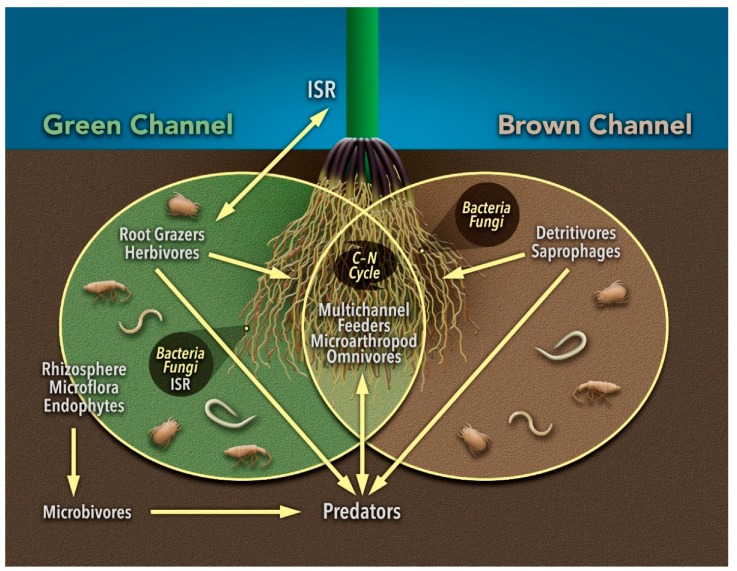
Carbon originates from primary producers either from root exudates of living plants (green channel) or degradation of detritus (brown channel). Collectively, these channels link above- and below-ground resource pools. Abbreviations defined as induced systemic resistance (ISR), carbon (C), and nitrogen (N). Figure credit: N. Sloff.

Living above- and below-ground plant material, root exudates, and detritus are major resource pools for soil organisms. Living plant-material and grazing organisms are sometimes described as the “green” feeding channel, whereas detritus and detritivores are described as the “brown” channel [103,104,105,106]. Hypotheses and models about interactions between microarthropods and soil health are typically limited to activities in one or the other energy channel (Figure 1). For example, soil microarthropods are often assigned to distinct trophic groups, including herbivores, detritivores, microbivores and predators [107]. However, more recently, it has been recognized that microarthropods function within complex food chains and represent a continuum of trophic behavior with a high degree of omnivory [108,109], and multi-channel feeding across energy sources is considered to be a distinguishing characteristic of soil food webs [105].

Collembolans and mites are very diverse in their feeding behavior and their trophic level spans from primary and secondary decomposers, feeding, predominantly on litter or fungi, to predators (feeding predominantly on nematodes and microarthropods) [86,110,111,112]. Stable isotope analysis of a below-ground food web in maize indicated that several microarthropod species form a feeding behavior gradient rather than a discrete trophic group, and that predators largely functioned as trophic level omnivores [113]. There is evidence that multi-channel feeding in soil probably contributes to some level of mortality of pest organisms. For example, ^15^N:^14^N isotope ratios for 20 collembolan species revealed that trophic behavior spans a continuum across three trophic levels from herbivory to primary and secondary decomposition and encompasses a gradual shift in diet from detrital to more microbial [110]. Furthermore, stable isotope analyses indicate that several putative detritivorous oribatid mites, *Liacarus subterraneus*, *Platynothrus peltifer* and *S. magnus,* are more likely to live as predators on nematodes or scavengers than as detritivores [85]. Microarthropods traditionally considered mycophagous may also be nematophagous [114]. For example, the collembolan, *Folsomia candida*, will preferentially feed upon the nematode, *Caenorhabditis elegans*, rather than fungi [115].

Multi-channel omnivory likely occurs at most or all food chain levels and may regulate the structure and function of soil food webs. The coupling of channels adds stability to soil food webs [116] when nutrients move through one channel at a different rate than the other [117]. Impacts of predation may skip a link in the food chain creating a trophic cascade [19]. For example, a mite feeds on a collembolan, which allows the fungal prey to increase its contribution to decomposition [75].

### 3.7. Climate Change

Soil fauna may be greatly affected by climate change, but the evidence of this is underrepresented in the literature [17,118,119]. Soils are a crucial part of climate change mitigation and adaptation, and it is critical that soil fauna are included in management decisions that affect global carbon budgets. Global carbon models depend on the sequestration of carbon in soils. Previously, recalcitrant materials such as humus were considered inert because of the high activation energy of oxidative enzymes, but rising temperatures may make that argument less plausible [120]. The mechanisms associated with structure and protection of SOM are still unresolved. Gram-negative bacteria produce continuous supplies of glucose to soil that ‘prime’ decomposition, rendering humus as vulnerable as labile carbon [121]. Soil fauna may modify this process by affecting the amount and quality of dissolved organic matter (DOM) entering the soil from decomposing litter. Fauna that consume food with much higher C:N ratios than in their own bodies contribute to the release of DOM into the soil [122]. Omnivory by microarthropods could play a role in driving stable trophic dynamics of soil food webs [105].

Accurate predictions of climate change effects require a better understanding of the complex interactions of environmental factors on soil faunal community dynamics. Climate change models predict increased frequency of temperature and precipitation extremes [123]. Rising temperatures impact below-ground ecosystems and can alter relationships among plants, soil microorganisms, and fauna. These changes differentially affect the quality of plant litter entering the soil and soil microarthropod taxa, with cascading effects on decomposition rate [124,125]. Several attributes of soil fauna respond to increased concentrations of CO_2_ but no general pattern of response has been identified [126]. For example, populations of Collembola increase with moisture and decrease with elevated temperatures, whereas oribatid mites are insensitive to elevated temperature and decrease with moisture [127,128]. However, this pattern is variable. For example, mesostigmatid mites are sensitive to warming temperatures similar to collembolans [129]. Some collembolans, including Sminthuridae, Tomoceridae, and Entomobryidae, respond in an inverse manner to rainfall and soil depth compared with Gnaphosidae spiders [130]. Although it depends on moisture, increased temperatures at high latitude will attract migration of the hitherto absent large comminuters (e.g., earthworms). Large comminuters potentially augment the activity of microarthropods resulting in a significant increase in decomposition rates [124]. Climate change and climate variability present a challenge to ecologically, economically, and socially sustainable land management. Managing soils to increase soil health and carbon sequestration will provide a more complete picture of how to manage the impacts of climate change [131].

## 4. Conclusions

Soil fauna are key components of soil health and sustainability. Soil scientists and microbial ecologists often acknowledge the role of microorganisms in decomposition and mineralization cycles but have paid less attention to the roles of micro- and meso-invertebrates [132]. Microarthropods affect SOM directly by fragmenting detritus, indirectly by affecting microbial activity, and influence fluxes between various SOM pools by multi-channel feeding. These relationships occur at local scales but how they translate to regional or global scales is unknown [133]. The response of microarthropods to disturbance is variable because of many contributing factors, including taxonomic and functional identity, type of disturbance, other soil biota, soil chemical and physical characteristics, and other site factors. We strongly agree with Grandy et al. [134] and Soong and Nielsen [135], who argue for including soil fauna in food web and biogeochemical models to increase our understanding of the function of soil. Understanding the impacts of environmental changes on the relationships between soil fauna and ecological function are of critical importance to the future sustainability of global food and fiber production systems, especially in the context of global climate change [1,17].

## References

[1] Wardle D.A., Bardgett R.D., Klironomos J.N., Setala H., van der Putten W.H., Wall D.H. (2004). Ecological linkages between aboveground and belowground biota. Science.

[2] Garcia-Palacios P., Maestre F.T., Kattge J., Wall D.H. (2013). Climate and litter quality differently modulate the effects of soil fauna on litter decomposition across biomes. Ecol. Lett..

[3] Siddiky M.R.K., Schaller J., Caruso T., Rillig M.C. (2012). Arbuscular mycorrhizal fungi and collembola non-additively increase soil aggregation. Soil Biol. Biochem..

[4] Wolters V. (2000). Invertebrate control of soil organic matter stability. Biol. Fert. Soils.

[5] Six J., Bossuyt H., Degryze S., Denef K. (2004). A history of research on the link between (micro) aggregates, soil biota, and soil organic matter dynamics. Soil Till. Res..

[6] Berendsen R.L., Pieterse C.M., Bakker P.A. (2012). The rhizosphere microbiome and plant health. Trends Plant Sci..

[7] Briones M.J.I. (2018). The serendipitous value of soil fauna in ecosystem functioning: The unexplained explained. Front. Environ. Sci..

[8] NRCS Soil Health. https://www.nrcs.usda.gov/wps/portal/nrcs/main/national/soils/health/.

[9] Lavelle P., Decaens T., Aubert M., Barot S., Blouin M., Bureau F., Margerie P., Mora P., Rossi J.P. (2006). Soil invertebrates and ecosystem services. Eur. J. Soil Biol..

[10] Culliney T. (2013). Role of arthropods in maintaining soil fertility. Agriculture.

[11] Wagg C., Bender S.F., Widmer F., van der Heijden M.G. (2014). Soil biodiversity and soil community composition determine ecosystem multifunctionality. Proc. Natl. Acad. Sci. USA.

[12] Seastedt T.R. (1984). The role of microarthropods in decomposition and mineralization processes. Annu. Rev. Entomol..

[13] Neher D.A. (1999). Soil community composition and ecosystem processes—Comparing agricultural ecosystems with natural ecosystems. Agroforest. Syst..

[14] Coleman D.C., Callaham M.A.J., Crossley D.A.J. (2018). Fundamentals of Soil Ecology.

[15] Castro-Huerta R.A., Falco L.B., Sandler R.V., Coviella C.E. (2015). Differential contribution of soil biota groups to plant litter decomposition as mediated by soil use. PeerJ.

[16] Kampichler C., Bruckner A. (2009). The role of microarthropods in terrestrial decomposition: A meta-analysis of 40 years of litterbag studies. Biol. Rev..

[17] Nielsen U.N., Wall D.H., Six J. (2015). Soil biodiversity and the environment. Annu. Rev. Env. Resour..

[18] Brussaard L., Pulleman M.M., Ouedraogo E., Mando A., Six J. (2007). Soil fauna and soil function in the fabric of the food web. Pedobiologia.

[19] Moore J.C., Walter D.E., Hunt H.W. (1988). Arthropod regulation of micro- and mesobiota in below-ground detrital food webs. Annu. Rev. Entomol..

[20] Feeny P. (1976). Plant apparency and chemical defense. Biochemical Interaction between Plants and Insects.

[21] Root R.B. (1973). Organization of a plant-arthropod association in simple and diverse habitats: The fauna of collards (*Brassica oleracea*). Ecol. Monogr..

[22] Bardgett R.D., Wardle D.A. (2010). Aboveground-Belowground Linkages: Biotic Interactions, Ecosystem Processes.

[23] Bezemer T.M., van Dam N.M. (2005). Linking aboveground and belowground interactions via induced plant defenses. Trends Ecol. Evol..

[24] Van der Putten W.H., Vet L.E.M., Harvey J.A., Wackers F.L. (2001). Linking above- and belowground multitrophic interactions of plants, herbivores, pathogens, and their antagonists. Trends Ecol. Evol..

[25] Erb M., Lenk C., Degenhardt J., Turlings T.C. (2009). The underestimated role of roots in defense against leaf attackers. Trends Plant Sci..

[26] Moreau G., Eveleigh E.S., Lucarotti C.J., Quiring D.T. (2006). Ecosystem alteration modifies the relative strengths of bottom-up and top-down forces in a herbivore population. J. Anim. Ecol..

[27] Morris W.F., Hufbauer R.A., Agrawal A.A., Bever J.D., Borowicz V.A., Gilbert G.S., Maron J.L., Mitchell C.E., Parker I.M., Power A.G. (2007). Direct and interactive effects of enemies and mutualists on plant performance: A meta-analysis. Ecology.

[28] Phelan P.L. (1997). Soil-management history and the role of plant mineral balance as a determinant of maize susceptibility to the European corn borer. Biol. Agric. Hortic..

[29] Bezemer T.M., Fountain M.T., Barea J.M., Christensen S., Dekker S.C., Duyts H., van Hal R., Harvey J.A., Hedlund K., Maraun M. (2010). Divergent composition but similar function of soil food webs of individual plants: Plant species and community effects. Ecology.

[30] Ruf A., Kuzyakov Y., Lopatovskaya O. (2006). Carbon fluxes in soil food webs of increasing complexity revealed by C-14 labelling and C-13 natural abundance. Soil Biol. Biochem..

[31] Drigo B., Pijl A.S., Duyts H., Kielak A., Gamper H.A., Houtekamer M.J., Boschker H.T.S., Bodelier P.L.E., Whiteley A.S., van Veen J.A. (2010). Shifting carbon flow from roots into associated microbial communities in response to elevated atmospheric CO_2_. Proc. Natl. Acad. Sci. USA.

[32] Ostle N., Briones M.J.I., Ineson P., Cole L., Staddon P., Sleep D. (2007). Isotopic detection of recent photosynthate carbon flow into grassland rhizosphere fauna. Soil Biol. Biochem..

[33] Bonkowski M., Clarholm M. (2012). Stimulation of plant growth through interactions of bacteria and protozoa: Testing the auxiliary microbial loop hypothesis. Acta Protozool..

[34] Chamberlain P.M., McNamara N.P., Chaplow J., Stott A.W., Black H.I.J. (2006). Translocation of surface litter carbon into soil by Collembola. Soil Biol. Biochem..

[35] Wickings K., Grandy A.S. (2011). The oribatid mite *Scheloribates moestus* (Acari: Oribatida) alters litter chemistry and nutrient cycling during decomposition. Soil Biol. Biochem..

[36] Kinnebrew E., Palawat K., Neher D.A., Galford G.L. (2019). Detritivores’ contributions to carbon cycling: Implications for ecosystem servicese and agricultural benefits. Environ. Res. Lett..

[37] Bradford M.A., Berg B., Maynard D.S., Wieder W.R., Wood S.A. (2016). Understanding the dominant controls on litter decomposition. J. Ecol..

[38] Dominati E., Patterson M., Mackay A. (2010). A framework for classifying and quantifying the natural capital and ecosystem services of soils. Ecol. Econ..

[39] Filser J. (2002). The role of Collembola in carbon and nitrogen cycling in soil. Pedobiologia.

[40] Frouz J. (2018). Effects of soil macro- and mesofauna on litter decomposition and soil organic matter stabilization. Geoderma.

[41] Kuťáková E., Cesarz S., Munzbergova Z., Eisenhauer N. (2018). Soil microarthropods alter the outcome of plant-soil feedback experiments. Sci. Rep..

[42] Teuben A., Verhoef H.A. (1992). Direct contribution by soil arthropods to nutrient availability through body and faecal nutrient content. Biol. Fert. Soils.

[43] Rodríguez I., Crespo G., Fraga S., Rodríguez C., Prieto D. (2003). Activity of the mesofauna and the macrofauna in dung patches during their decomposition process. Cuban J. Agric. Sci..

[44] Beanland L., Phelan P.L., Salminen S. (2003). Micronutrient interactions on soybean growth and the developmental performance of three insect herbivores. Environ. Entomol..

[45] Busch J.W., Phelan P.L. (1999). Mixture models of soybean growth and herbivore performance in response to nitrogen–sulphur–phosphorous nutrient interactions. Ecol. Entomol..

[46] Chaboussou F. (2004). Healthy Crops: A New Agricultural Revolution.

[47] Waring G.L., Cobb N.S., Bernays E.A. (1992). The impact of plant stress on herbivore population dynamics. Insect-Plant Interactions.

[48] Alyokhin A., Atlihan R. (2005). Reduced fitness of the Colorado potato beetle (Coleoptera: Chrysomelidae) on potato plants grown in manure-amended soil. Environ. Entomol..

[49] Boiteau G., Lynch D.H., Martin R.C. (2008). Influence of fertilization on the Colorado potato beetle, *Leptinotarsa decemlineata*, in organic potato production. Environ. Entomol..

[50] Pangesti N., Pineda A., Pieterse C.M., Dicke M., van Loon J.J. (2013). Two-way plant mediated interactions between root-associated microbes and insects: From ecology to mechanisms. Front. Plant Sci..

[51] Papadopoulou G.V., van Dam N.M. (2017). Mechanisms and ecological implications of plant-mediated interactions between belowground and aboveground insect herbivores. Ecol. Res..

[52] Pieterse C.M., Van der Does D., Zamioudis C., Leon-Reyes A., Van Wees S.C. (2012). Hormonal modulation of plant immunity. Annu. Rev. Cell Dev. Biol..

[53] Pineda A., Zheng S.J., van Loon J.J., Pieterse C.M., Dicke M. (2010). Helping plants to deal with insects: The role of beneficial soil-borne microbes. Trends Plant Sci..

[54] Van Dam N.M., Heil M. (2011). Multitrophic interactions below and above ground: En route to the next level. J. Ecol..

[55] Briones M.J. (2014). Soil fauna and soil functions: A jigsaw puzzle. Front. Environ. Sci..

[56] Bukovinszky T., van Veen F.J.F., Jongema Y., Dicke M. (2008). Direct and indirect effects of resource quality on food web structure. Science.

[57] Kaplan I., Halitschke R., Kessler A., Rehill B.J., Sardanelli S., Denno R.F. (2008). Physiological integration of roots and shoots in plant defense strategies links above- and belowground herbivory. Ecol. Lett..

[58] Soler R., Van der Putten W.H., Harvey J.A., Vet L.E., Dicke M., Bezemer T.M. (2012). Root herbivore effects on aboveground multitrophic interactions: Patterns, processes and mechanisms. J. Chem. Ecol..

[59] Wurst S. (2013). Plant-mediated links between detritivores and aboveground herbivores. Front. Plant Sci..

[60] Mithöfer A., Boland W. (2012). Plant defense against herbivores: Chemical aspects. Annu. Rev. Plant Biol..

[61] Schuman M.C., Baldwin I.T. (2016). The layers of plant responses to insect herbivores. Annu. Rev. Entomol..

[62] Balbi V., Devoto A. (2008). Jasmonate signalling network in *Arabidopsis thaliana*: Crucial regulatory nodes and new physiological scenarios. New Phytol..

[63] De Moraes C.M., Mescher M.C., Tumlinson J.H. (2001). Caterpillar-induced nocturnal plant volatiles repel conspecific females. Nature.

[64] Turlings T.C., Tumlinson J.H., Lewis W.J. (1990). Exploitation of herbivore-induced plant odors by host-seeking parasitic wasps. Science.

[65] Robert-Seilaniantz A., Grant M., Jones J.D.G. (2011). Hormone crosstalk in plant disease and defense: More than just jasmonate-salicylate antagonism. Annu. Rev. Phytopathol..

[66] Hiltpold I., Erb M., Robert C.A.M., Turlings T.C.J. (2011). Systemic root signalling in a belowground, volatile-mediated tritrophic interaction. Plant Cell Environ..

[67] Rasmann S., Turlings T.C.J. (2008). First insights into specificity of belowground tritrophic interactions. Oikos.

[68] Walling L.L. (2000). The myriad plant responses to herbivores. J. Plant Growth Regul..

[69] Rodriguez P.A., Rothballer M., Chowdhury S.P., Nussbaumer T., Gutjahr C., Falter-Braun P. (2019). Systems biology of plant-microbiome interactions. Mol. Plant.

[70] Thaler J.S., Humphrey P.T., Whiteman N.K. (2012). Evolution of jasmonate and salicylate signal crosstalk. Trends Plant Sci..

[71] Ourry M., Lebreton L., Chaminade V., Guillerm-Erckelboudt A.Y., Herve M., Linglin J., Marnet N., Ourry A., Paty C., Poinsot D. (2018). Influence of belowground herbivory on the dynamics of root and rhizosphere microbial communities. Front. Ecol. Evol..

[72] Endlweber K., Ruess L., Scheu S. (2009). Collembola switch diet in presence of plant roots thereby functioning as herbivores. Soil Biol. Biochem..

[73] Janoušková M., Kohout P., Moradi J., Doubková P., Frouz J., Vosolsobě S., Rydlová J. (2018). Microarthropods influence the composition of rhizospheric fungal communities by stimulating specific taxa. Soil Biol. Biochem..

[74] Ngosong C., Gabriel E., Ruess L. (2014). Collembola grazing on arbuscular mycorrhiza fungi modulates nutrient allocation in plants. Pedobiologia.

[75] Hedlund K., Öhrn M.S. (2000). Tritrophic interactions in a soil community enhance decomposition rates. Oikos.

[76] Maraun M., Martens H., Migge S., Theenhaus A., Scheu S. (2003). Adding to ‘the enigma of soil animal diversity’: Fungal feeders and saprophagous soil invertebrates prefer similar food substrates. Eur. J. Soil Biol..

[77] Losey J.E., Vaughan M. (2006). The economic value of ecological services provided by insects. Bioscience.

[78] Naranjo S.E., Ellsworth P.C., Frisvold G.B. (2015). Economic value of biological control in integrated pest management of managed plant systems. Annu. Rev. Entomol..

[79] Berg M.P., Stoffer M., van den Heuvel H.H. (2004). Feeding guilds in Collembola based on digestive enzymes. Pedobiologia.

[80] Köehler H.H. (1997). Mesostigmata (Gamasina, Uropodina), efficient predators in agroecosystems. Agric. Ecol. Ecosyst..

[81] Read D.S., Sheppard S.K., Bruford M.W., Glen D.M., Symondson W.O.C. (2006). Molecular detection of predation by soil micro-arthropods on nematodes. Mol. Ecol..

[82] Brust G.E. (1990). Effects of below-ground predator–weed interactions on damage to peanut by southern corn rootworm (Coleoptera: Chrysomelidae). Environ. Entomol..

[83] Welbourne W.C., Hoy M.A., Cunningham G.L., Knutsen L. (1983). Potential use of trombidioid and erythraeoid mites as biological control agents of insect pests. Biological Control of Pests by Mites.

[84] Brust G.E., House G.J. (1988). A study of *Tyrophagus putrescentiae* (Acari: Acaridae) as a facultative predator of southern corn rootworm eggs. Exp. Appl. Acarol..

[85] Heidemann K., Scheu S., Ruess L., Maraun M. (2011). Molecular detection of nematode predation and scavenging in oribatid mites: Laboratory and field experiments. Soil Biol. Biochem..

[86] Kaneda S., Kaneko N. (2008). Collembolans feeding on soil affect carbon and nitrogen mineralization by their influence on microbial and nematode activities. Biol. Fert. Soils.

[87] Sayre R.M., Walter D.E. (1991). Factors affecting the efficacy of natural enemies of nematodes. Annu. Rev. Phytopathol..

[88] Schneider K., Migge S., Norton R.A., Scheu S., Langel R., Reineking A., Maraun M. (2004). Trophic niche differentiation in soil microarthropods (Oribatida, Acari): Evidence from stable isotope ratios (^15^N/^14^N). Soil Biol. Biochem..

[89] Doncaster C.C., Murphy P.W. (1957). A culture method for soil meiofauna and its application to the study of nematode predators 1. Nematologica.

[90] Chen B., Snider R.J., Snider R.M. (1995). Food preference and effects of food type on the life history of some soil Collembola. Pedobiologia.

[91] Polis G.A., Strong D.R. (1996). Food web complexity and community dynamics. Am. Nat..

[92] Chen B.R., Wise D.H. (1999). Bottom-up limitation of predaceous arthropods in a detritus-based terrestrial food web. Ecology.

[93] Rypstra A.L., Marshall S.D. (2005). Augmentation of soil detritus affects the spider community and herbivory in a soybean agroecosystem. Entomol. Exp. Appl..

[94] Garratt M.P.D., Wright D.J., Leather S.R. (2011). The effects of farming system and fertilisers on pests and natural enemies: A synthesis of current research. Agric. Ecol. Ecosyst..

[95] Wise D.H., Snyder W.E., Tuntibunpakul P., Halaj J. (1999). Spiders in decomposition food webs of agroecosystems: Theory and evidence. J. Arachnol..

[96] Muñoz-Cárdenas K., Ersin F., Pijnakker J., van Houten Y., Hoogerbrugge H., Leman A., Pappas M.L., Duarte M.V.A., Messelink G.J., Sabelis M.W. (2017). Supplying high-quality alternative prey in the litter increases control of an above-ground plant pest by a generalist predator. Biol. Control.

[97] Bilde T., Axelsen J.A., Toft S. (2000). The value of Collembola from agricultural soils as food for a generalist predator. J. Appl. Ecol..

[98] Halaj J., Wise D.H. (2002). Impact of a detrital subsidy on trophic cascades in a terrestrial grazing food web. Ecology.

[99] McNabb D.M., Halaj J., Wise D.H. (2001). Inferring trophic positions of generalist predators and their linkage to the detrital food web in agroecosystems: A stable isotope analysis. Pedobiologia.

[100] Koss A.M., Snyder W.E. (2005). Alternative prey disrupt biocontrol by a guild of generalist predators. Biol. Control.

[101] Symondson W.O., Cesarini S., Dodd P.W., Harper G.L., Bruford M.W., Glen D.M., Wiltshire C.W., Harwood J.D. (2006). Biodiversity vs. biocontrol: Positive and negative effects of alternative prey on control of slugs by carabid beetles. Bull. Entomol. Res..

[102] Birkhofer K., Wise D.H., Scheu S. (2008). Subsidy from the detrital food web, but not microhabitat complexity, affects the role of generalist predators in an aboveground herbivore food web. Oikos.

[103] Scheunemann N., Digel C., Scheu S., Butenschoen O. (2015). Roots rather than shoot residues drive soil arthropod communities of arable fields. Oecologia.

[104] Wolkovich E.M., Allesina S., Cottingham K.L., Moore J.C., Sandin S.A., de Mazancourt C. (2014). Linking the green and brown worlds: The prevalence and effect of multichannel feeding in food webs. Ecology.

[105] Wolkovich E.M. (2016). Reticulated channels in soil food webs. Soil Biol. Biochem..

[106] Zou K., Thébault E., Lacroix G., Barot S. (2016). Interactions between the green and brown food web determine ecosystem functioning. Funct. Ecol..

[107] Bardgett R.D. (2005). The Biology of Soil: A Community and Ecosystem Approach.

[108] Digel C., Curtsdotter A., Riede J., Klarner B., Brose U. (2014). Unravelling the complex structure of forest soil food webs: Higher omnivory and more trophic levels. Oikos.

[109] Glavatska O., Muller K., Butenschoen O., Schmalwasser A., Kandeler E., Scheu S., Totsche K.U., Ruess L. (2017). Disentangling the root- and detritus-based food chain in the micro-food web of an arable soil by plant removal. PLoS ONE.

[110] Chahartaghi M., Langel R., Scheu S., Ruess L. (2005). Feeding guilds in Collembola based on nitrogen stable isotope ratios. Soil Biol. Biochem..

[111] Klarner B., Maraun M., Scheu S. (2013). Trophic diversity and niche partitioning in a species rich predator guild–Natural variations in stable isotope ratios (^13^C/^12^C, ^15^N/^14^N) of mesostigmatid mites (Acari, Mesostigmata) from Central European beech forests. Soil Biol. Biochem..

[112] Schneider K.C.R., Scheu S., Maraun M. (2004). Feeding biology of oribatid mites: A minireview. Phytophaga.

[113] Albers D., Schaefer M., Scheu S. (2006). Incorporation of plant carbon into the soil animal food web of an arable system. Ecology.

[114] Walter D.E. (1987). Trophic behavior of mycophagous microarthropods. Ecology.

[115] Lee Q., Widden P. (1996). *Folsomia candida*, a “fungivorous” collembolan, feeds preferentially on nematodes rather than soil fungi. Soil Biol. Biochem..

[116] Moore J.C., Berlow E.L., Coleman D.C., de Ruiter P.C., Dong Q., Hastings A., Johnson N.C., McCann K.S., Melville K., Morin P.J. (2004). Detritus, trophic dynamics and biodiversity. Ecol. Lett..

[117] Rooney N., McCann K.S. (2012). Integrating food web diversity, structure and stability. Trends Ecol. Evol..

[118] McKenzie D.C. (2013). Visual soil examination techniques as part of a soil appraisal framework for farm evaluation in Australia. Soil Till. Res..

[119] Van der Putten W.H., Macel M., Visser M.E. (2010). Predicting species distribution and abundance responses to climate change: Why it is essential to include biotic interactions across trophic levels. Philos. Trans. R. Soc. B-Biol. Sci..

[120] Dungait J.A.J., Hopkins D.W., Gregory A.S., Whitmore A.P. (2012). Soil organic matter turnover is governed by accessibility not recalcitrance. Glob. Chang. Biol..

[121] Ekelund F., Saj S., Vestergård M., Bertaux J., Mikola J. (2009). The “soil microbial loop” is not always needed to explain protozoan stimulation of plants. Soil Biol. Biochem..

[122] Osler G.H., Sommerkorn M. (2007). Toward a complete soil C and N cycle: Incorporating the soil fauna. Ecology.

[123] Field C.B., Barros V., Stocker T.F., Dahe Q., ICPP (2012). Managing the Risks of Extreme Events and Disasters to Advance Climate Change Adaptation: Special Report of the Intergovernmental Panel on Climate Change.

[124] Aerts R. (2006). The freezer defrosting: Global warming and litter decomposition rates in cold biomes. J. Ecol..

[125] Taylor A.R., Schroter D., Pflug A., Wolters V. (2004). Response of different decomposer communities to the manipulation of moisture availability: Potential effects of changing precipitation patterns. Glob. Chang. Biol..

[126] Coûteaux M.M., Bolger T. (2000). Interactions between atmospheric CO_2_ enrichment and soil fauna. Plant Soil.

[127] Darby B.J., Neher D.A., Housman D.C., Belnap J. (2011). Few apparent short-term effects of elevated soil temperature and increased frequency of summer precipitation on the abundance and taxonomic diversity of desert soil micro-and meso-fauna. Soil Biol. Biochem..

[128] Ferguson S.H., Joly D.O. (2002). Dynamics of springtail and mite populations: The role of density dependence, predation, and weather. Ecol. Entomol..

[129] Sjursen H., Michelsen A., Jonasson S. (2005). Effects of long-term soil warming and fertilisation on microarthropod abundances in three sub-arctic ecosystems. Appl. Soil Ecol..

[130] Lensing J.R., Todd S., Wise D.H. (2005). The impact of altered precipitation on spatial stratification and activity-densities of springtails (Collembola) and spiders (Araneae). Ecol. Entomol..

[131] Coyle D.R., Nagendra U.J., Taylor M.K., Campbell J.H., Cunard C.E., Joslin A.H., Mundepi A., Phillips C.A., Callaham M.A. (2017). Soil fauna responses to natural disturbances, invasive species, and global climate change: Current state of the science and a call to action. Soil Biol. Biochem..

[132] Lavelle P. (2000). Ecological challenges for soil science. Soil Sci..

[133] Wall D.H., Bradford M.A., St John M.G., Trofymow J.A., Behan-Pelletier V., Bignell D.D.E., Dangerfield J.M., Parton W.J., Rusek J., Voigt W. (2008). Global decomposition experiment shows soil animal impacts on decomposition are climate-dependent. Glob. Chang. Biol..

[134] Grandy A.S., Wieder W.R., Wickings K., Kyker-Snowman E. (2016). Beyond microbes: Are fauna the next frontier in soil biogeochemical models?. Soil Biol. Biochem..

[135] Soong J.L., Nielsen U.N. (2016). The role of microarthropods in emerging models of soil organic matter. Soil Biol. Biochem..

